# Community health workers: challenges and vulnerabilities of Accredited Social Health Activists working in conflict-affected settings in the state of Assam, India

**DOI:** 10.1186/s12913-021-06780-y

**Published:** 2021-08-17

**Authors:** Preety R Rajbangshi, Devaki Nambiar, Aradhana Srivastava

**Affiliations:** 1 The George Institute for Global Health, 311-312, Third Floor, Elegance Tower,Plot No.8,Jasola District Centre, 10025 New Delhi, India; 2 Faculty of Medicine, University of New South Wales, Kensington, Australia; 3 Prasanna School of Public Health, Manipal Academy of Higher Education, Manipal, India; 4 World Food Programme, A-2, Poorvi Marg, Block A, Vasant Vihar, 110057 New Delhi, India

**Keywords:** Community health workers, Assam, India, Conflict

## Abstract

**Introduction:**

It is well acknowledged that India’s community health workers known as Accredited Social Health Activists (ASHA) are the bedrock of its health system. Many ASHAs are currently working in fragile and conflict-affected settings. No efforts have yet been made to understand the challenges and vulnerabilities of these female workers. This paper seeks to address this gap by bringing attention to the situation of ASHAs working in the fragile and conflict settings and how conflict impacts them and their work.

**Methods:**

Qualitative fieldwork was undertaken in four conflict-affected villages in two conflict-affected districts -Kokrajhar and Karbi Anglong of Assam state situated in the North-East region of India. Detailed account of four ASHAs serving roughly 4000 people is presented. Data transliterated into English were analysed by authors by developing a codebook using grounded theory and thematic organisation of codes.

**Results:**

ASHAs reported facing challenges in ensuring access to health services during and immediately after outbreaks of conflict. They experienced difficulty in arranging transport and breakdown of services at remote health facilities. Their physical safety and security were at risk during episodes of conflict. ASHAs reported hostile attitudes of the communities they served due to the breakdown of social relations, trauma due to displacement, and loss of family members, particularly their husbands.

**Conclusions:**

Conflict must be recognised as an important context within which community health workers operate, with greater policy focus and research devoted to understanding and addressing the barriers they face as workers and as persons affected by conflict.

## Introduction

India’s Community Health Workers (CHWs), known as Accredited Social Health Activists (ASHAs), are the bedrock of its health system. Nearly one million trained ASHAs are working as volunteers in the largest government-led CHW programme globally [1]. These workers receive performance-based incentives linked to different health programmes implemented by Indian states. ASHAs are female residents of the villages they serve; they are the interface between their communities and the health system. Their main role is to generate awareness in their communities about health and its determinants and support the delivery of primary health care [2]. Several evaluations of the ASHA programme have established their contribution towards improved awareness, behaviour change, and positive impact on the health and well-being of their communities [1–4]. Concurrently, an extensive global body of literature suggests and supports CHWs effectiveness in the delivery of primary healthcare programmes in different settings [5]. For example, several humanitarian organizations have established CHW programmes as a means to increase access to health services during and after humanitarian emergencies [5]. Conflict-affected health systems in countries like Afghanistan, Burkina Faso have also engaged CHWs to provide essential services under restrictive and sometimes dangerous situations [6]. In India, we found one study demonstrating the effectiveness of a lay health worker intervention in a rural conflict ridden district of Kashmir state [7], and another showing feasibility of community health worker supported tuberculosis patient tracing using GeneXpert in naxal conflict affected border areas of Chhattisgarh state [7, 8]. However, in neither case were ASHA workers involved even as they are employed in all of the conflict -affected regions of india, which include the erstwhile state of Jammu and Kashmir as well as six of the eight states in the northeast region.

While the current literature globally offers guidance on the effectiveness of CHWs and optimizing their performance, the challenges and vulnerabilities of CHWs working in fragile and conflict-affected settings remain inadequately understood within the field of human resources for health. Clearly, the need for such evidence is pressing [5]. In India, for example, a recent volume on conflict and heath inequities in India’s northeast region placed emphasis on absence of doctors and the challenge of informal care provision: the role of Commuity Health Workers (CHWs) [9], however, is under-explored in the academic literature.This paper seeks to address this gap by bringing attention to the situation of CHWs working in the fragile and conflict settings, and the impact of conflict on them and their work.

We present the findings from a larger study on women’s reproductive health access conducted in the conflict-affected state of Assam in the North-East region of India. Assam is a multiethnic and multilingual state with 31 million people [10]. It has been one of the most volatile and sensitive states in the region; for more than half a century, the state has witnessed conflicts ranging from insurgency for autonomy and domestic terrorism to ethnic violence [11]. There are several extremist outfits in the state aiming to achieve different objectives based on ethnicity and territory [11]. Currently, conflict in Assam is shaped by ethnic polarization and the assertion of community identities. This includes resistance to the settlement of perceived foreigners from Bangladesh [faced by Bengali origin Muslims], inter-tribal hostilities, and alienation of non-tribal migrant communities belonging to different states in India. While conflict is on the decline and confined to seven out of 33 districts of Assam, its effect is evident in these districts in the form of destruction of social infrastructure [12, 13].

This study was conducted in two conflict-affected settings – Kokrajhar and Karbi Anglong districts in Assam. The conflict in both the districts started with the struggle for a separate homeland for tribal communities: the Bodo in Kokrajhar and Karbi in Karbi Anglong district. This was followed by waves of ethnic conflict in both districts. The conflict in Kokrajhar is the oldest as it started in the late 1980s in the form of a violent movement for a separate homeland, continuing into the 1990 s until 2003 [12]. This was followed by waves of ethnic conflict from 1996 onwards between the tribal Bodo community and non-tribal ethnic groups – Santhal or Adivasi and Bengali origin Muslims, led by militant groups belonging to the Bodo community seeking to drive out non-Bodos and create ethnically more homogenous region known as Bodoland Territorial Council [12, 14]. The conflict in 2012 between Bodo and Bengali origin Muslim communities saw the largest internal displacement in post-independence India with 10 % increase in new displacement compared to previous year [15, 16].

In the past 25 years, Kokrajhar has seen eight waves of ethnic conflict [12, 17]. In line with Kokrajhar, the conflict in Karbi Anglong started with a demand for statehood in the 1990s which was followed by inter-tribal conflict led by militant groups representing different tribal communities [13]. Apart from repeated conflicts, both the districts are vulnerable to pervasive militant conflict. A study in Kokrajhar found that decades of conflict have weakened the social fabric and infrastructure of the district [12]. This has increased health systems fragility and weakened the provision of health services to the majority rural population.

In both districts, ASHAs have been pivotal in building linkages between the health systems and community and support access to public health services. The ASHAs in Kokrajhar and Karbi Anglong districts are among the first to be recruited and trained in India, as Assam was one of the 18 states where the ASHA programme was first launched in 2006 under National Rural Health Mission (presently known a National Health Mission) [18]. The training, responsibilities, incentive, and supervision system for ASHAs in the state was primarily based on the Federal Ministry of Health’s guidelines for the ASHA programme with modifications based on the state’s requirements and policies.

## Methods

A qualitative, descriptive study was conducted to understand the impact of conflict on reproductive health service delivery and utilization in conflict-affected districts. As a part of the larger qualitative study, in-depth interviews were conducted with health providers ranging from ASHAs to doctors and nurses rendering health services in the conflict-affected villages. The themes experiences and challenges of providing health services in conflict-affected aeas serving roughly 4,000 people in four villages ( each ASHA catering to roughly 1000 people), and their vulnerabilities emerged during our interviews with the ASHAs were analyzed and presented as case studies in this paper.

 Formal permissions and support from the state and district health system were obtained for the larger study. Consultations with the District Programme Management Unit in the respective district was held to select the block and villages affected by conflict. After selecting the villages, participants were contacted in advance by three-member research teams comprising of the lead author, field investigator, and translator. A mix of purposive and convenience sampling methods was used for in-depth interviews with participants. Before data collection, training was imparted to the field investigator and translators of the respective district by the lead author. The focus was to orient the team with an interview guide, informed consent procedure, and ethical considerations.

Participant information sheet detailing the purpose, methods, risk and benefits of participating in the study was explained and shared with each participant. Following this, informed written consent was sought from the participants. Interviews were conducted in either Assamese language or the local dialect, depending on each participant’s preference. The interviews in the Assamese language were carried out by the lead author and for all others, the help of a translator was taken. All interviews were conducted in ASHAs residence and audio-recorded after taking permission. Each interview lasted between 30 and 45 min and in many cases, even if there was one formal interview, multiple interactions were carried out with each participant. The lead author and field investigator took field notes after each day’s fieldwork and these were discussed with the translators for their inputs and reflections. All interview recordings were stored in a folder accessible only to the research team. Interviews conducted in the Assamese language were transcribed by the field investigator and transcribers were engaged to transliterate from local dialect to the English language to obtain verbatim transcripts. The lead author performed quality checks. All fieldnotes and transcripts were cleaned and entered into Atlas. Ti software version 8.

An inductive conceptual framework was developed for the larger study and this framework was used to develop a codebook using grounded theory [19]. The codebook was developed through coding using Atlas. Ti followed by a discussion among the three authors. Codes were reviewed and finalized, following which codes were organized thematically, and indexed to facilitate interpretation. We initially presented findings as narrative by thematic areas, but during peer review, were advised to re-organise our analysis by focusing on individual cases. Therefore, themes were nested under case studies.

## Results

We present excerpts from our interaction and interviews with four ASHAs. Each had a different profile relative to the community she served and highlighted specific challenges that in some cases pertained to delivering or linking communities to care, and in others, reflected the challenges and insecurities faced as a member of the community. The ASHAs we spoke to noted that their experiences were common to their colleagues working in conflict-affected areas of the district.

### Case 1: Junali and Displacement

Junali [name changed] was 47 years and belonged to Bodo community. Junali and her family were displaced from her native village, but was still covering 10 households in that location (she is formally listed as the ASHA from this village) and the areas where the people were residing after displacement. She noted that the distance affected her performance as she had to visit three places to mobilize communities for immunization and other services.

**Fig. 1:**
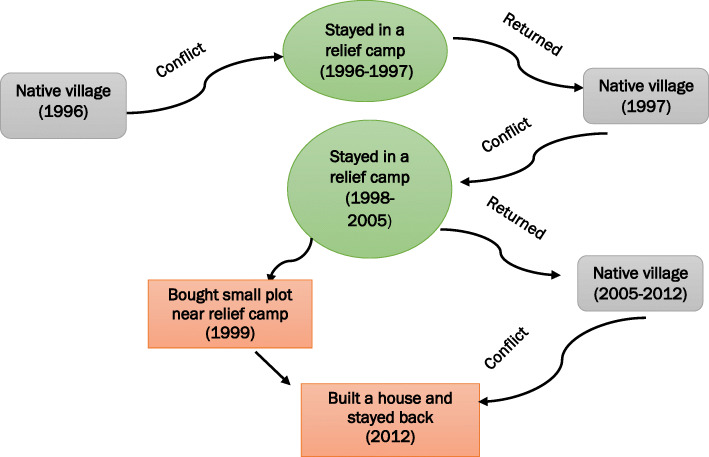
ASHA as a member of the community is susceptible to displacement. Source: Authors, based on Junali’s interview

While narrating the conflicts in the district, Junali reported that she and her family were internally displaced multiple times since 1996. Figure 1 summarizes the repeated displacement from 1996 to 2005. Junali noted that after the first conflict in 1996 her family stayed in a relief camp near block headquarter for one year before returning to the native village in 1998. After staying for a year in the village, they were again displaced for seven years. She and her family stayed in a relief camp. Out of fear of another displacement, her family bought a plot in the area close by to the relief camp but returned to their native village in 2005. However, in 2012, another round of conflict occurred and they had to leave their village again. The family decided to stay back and built a house in the newly purchased plot. Junali informed that almost all natives of her village had left their village and were staying in villages close by to her current residence.

It is noteworthy to mention that internal displacement – temporary or permanent- during the conflict was experienced by all four ASHAs. We observed that displacement had significantly affected their living conditions, particularly among ASHAs in Kokrajhar district. As they had experienced repetitive conflict since the 1990s. Like for other members of the community, displacement due to the conflict had led to the loss of farmland, livestock, and occupation of their family members. Jennifer (case 4), for example, had suffered permanent displacement after she decided to stay back in the area where relief camp was established as she believed that the current place was more secure than her native village. Displacement fragmented the community served by the ASHAs and also affected their perception of security risk, as they now had to traverse unfamiliar areas with unknown people in the course of their work.

During the discussion, Junali expressed her concern about returning home alone after completing the work at a health facility. This corroborated with Sarita’s (case 2) narrative where she reported working amidst fear.

*You understand sister, some of our women are in XY village...some in YY [name of the villages anonymised]. The way we live in the village, we feel scared when we accompany [pregnant women] for delivery and then we have to go the [health facility] office, how will I come [home] alone.*

### Case 2: Sarita on rebuilding trust in the face of practical challenges

Sarita [name changed] a 47 years old woman belonged to Koch-Rajbangshi community. She was serving communities belonging to three ethnic groups – Adivasi, Koch-Rajbangshi and Bengali origin Muslims. A majority of the people in the village belonged to Bengali origin Muslim community. The narrative presented below is from 2012 conflict between Bodo and Bengali origin Muslim communities.

A major challenge during the conflict was the breakdown of health services at the peripheral health facilities such as SC and PHC providing primary health care. Many times, ASHAs were left with the option of accessing the district hospital, which was already far away from their village and all the more difficult to reach at the time of conflict. We found that there was no support mechanism to ease the difficulty faced by ASHAs in ensuring access to health facilities during the conflict. It was left to ASHAs to arrange transportation for reaching the health facility. We noted that their challenges remained unaddressed.

Sarita also noted that women belonging to Bengali origin Muslim community were inhibited in reaching her for health advice although her community ( Koch – Rajbangshi) was not involved in the conflict. She further mentioned that those affected – the Bengali origin Muslim community - believed that her community conspired with the Bodo community in burning their houses. As a result, she noted, this community stopped trusting her. It is noteworthy to mention that ASHAs were also vulnerable to the hostile attitudes of the community and faced threats when the trust was lost between communities.

*It affected me as [Bengali speaking] Muslims did not believe me. The conflict was between Bodo and Muslim communities but the Muslim thought that my community was also involved in burning their houses. They kept to themselves and stopped coming to me for pregnancy care. The young Muslim boys were furious during the time of conflict and they tried to threaten me but I calmly explained to them. Now things have improved and Muslims in my area love me.*

We found that ethnic conflict in the districts had broken the social fabric in the community, in turn affecting ASHAs and their performance. Non-acceptance of ASHAs belonging to opposing ethnic communities was an articulated concern, particularly when the population catered by an ASHA comprised of people belonging to a different ethnic community. Even though Sarita did not mention her fear specifically, it was evident during our fieldwork that there was hesitation to visit hamlets belonging to certain communities, particularly when ASHA and the villagers belonged to conflicting communities. Notwithstanding the above, it is important to note that the inhibition and lack of trust were declining, due to long period of relative peace following the last conflict. We observed at the time of interview that women belonging to the Bengali origin Muslim community visited Sarita at her home.

Maintaining and rebuilding trust was particularly difficult owing to a number of practical challenges faced in conflict situations. Sarita pointed out the great difficulty faced by all ASHAs when the drivers of 108 emergency or 102 ambulatory services (implemented by the State National Health Mission) would refuse services due to fear and the family members of the pregnant woman would expect the ASHA to arrange transportation.

**Fig. 2:**
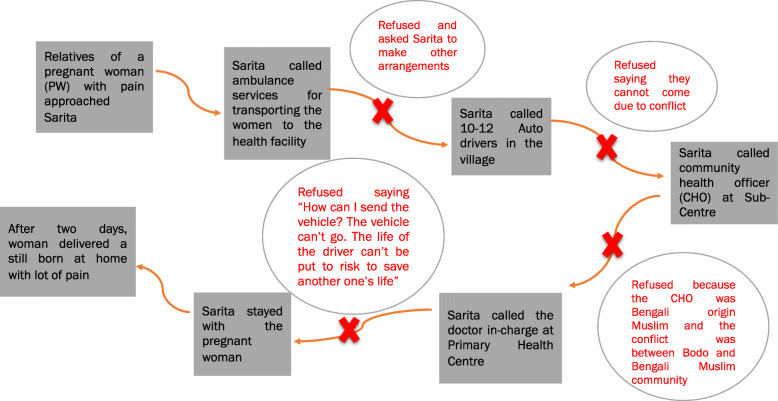
ASHA’s narrative regarding delivery services during the conflict. Source: Authors, based on Sarita’s interview

Figure 2 describes challenges faced by Sarita, as narrated by her, during the 2012 conflict where the family members of a pregnant woman approached and informed her that labour pain had started. She went with them and saw that the woman was in pain. Sarita immediately called 108 ambulance services but they refused to send a vehicle and asked her to make other arrangements. She even tried contacting drivers of private vehicles in the village but they too refused. In the meantime, she also contacted the Community Health Officer (CHO) at the sub-center (SC) and Medical Officer (In-charge) at the Primary Health Center (PHC) but they too could not help her. With no option left, the delivery was conducted at home: the woman suffered pain for two days and gave birth to a stillborn.

This difficulty in arranging transportation remained even months after the conflict episode and was reportedly due to pervasive fear among the drivers – both in the public and private sectors. Another such instance was shared by Sarita where she had to take a pregnant woman 15 days after the conflict had ended. That day, both 108 emergency service and 102 ambulances refused. She tried contacting private vehicles and luckily one auto-driver agreed to drive them to the health facility. However, while returning, as the driver was alone, army personnel stopped and beat him up for driving at the time of conflict. Because of this incident, the drivers were afraid and one auto-driver placed a condition that he would take pregnant woman only if Sarita returned with him and did not stay in the health facility along with the pregnant woman. This condition placed her in a dilemma as the health system expected her to be present during delivery and if she were to refuse the driver, this would affect his willingness to transport pregnant women in the future.

She further noted that the situation after conflict remained volatile for a few weeks, but she had accompanied pregnant women to the health facility for delivery by ignoring her fear: “*we had to take them [pregnant women] even if we were scared to travel on the road as we had no other options.”*

### Case 3: Anita and the tribulations of duty on the frontline

Anita [name changed] was 50 years old and belonged to Bodo community and the villages under her catchment areas had people belonging to her community. Anita and her family suffered temporary displacement during 1996 and 1998 conflict and stayed in relief camps. Anita recalled the challenges of living in a relief camp, such as receiving only small quantities of rice and pulses for food, which was not enough to avert hunger in her family. Recalling the conditions, Anita reported that people belonging to different ethnic communities were living peacefully prior to the conflicts. At the same time ,she acknowledged the role of peace building efforts and noted that fear between communities has reduced due to peace meetings organized by village heads in conflict-affected villages, and attended by communities engaged in conflict.

*“What comes to my mind is – we used to live and eat in harmony before, why did it [ conflict] happen, this thought keeps bothering me. It shouldn’t have happened, I don’t know much, but shouldn’t have happened, I keep thinking that”.*

While narrating the conflict of 2012, Anita noted that at the time of conflict she was engaged in a relief camp and supported the health staff in providing health services. According to Anita, all staff including ASHAs were expected to be available to provide services in relief camps, but there was no provision to pay for the work done by her and other ASHAs. She felt that it was her duty to provide health services; but also noted that she knew that the health system had funds for her work, but did not provide compensation. There was a feeling that ASHAs labour was not recognized.

*"We have to be bold and leave our home and family to help them [health system]. The Sir’s [doctors and district health officials] only tell us to do the work but don’t think about us even once. They need to look at our pain and problems…We work so hard at the relief camps, leaving our home and families, for such a long time. Even if we are afraid, we have to be bold and yet we don’t get any incentives or salary. We are expected to do the work for free. But we don’t back out and do our jobs as soon as we get our orders [from health officials]."*

Remunerations due for the services rendered become more uncertain in a conflict situation, even as the responsibility to ensure continuity of community health services increases the work pressure on the frontline workers. This creates a sense of despondency among the ASHAs, which is reflected in Anita’s statement. But their commitment drives them on.

Anita further noted that fear prevailed even after the conflict but it did not deter her from going to work .*“I went on my own to work when the conflict was over. Earlier [ during the conflict] nothing was done during the conflict. It was difficult to stay there even. There was a fear like- some miscreants will just appear from somewhere.”*

### Case 4: Jennifer and loss of family members

Jennifer [name changed] was 50 years old and belonged to the Adivasi community. A widow who lost her husband in 1996 conflict as he went missing. Jeniffer was the sole earning member of the family and was supporting her youngest daughter’s education by working as an ASHA and operating a small shop in the village. She had also adopted an orphaned boy whom she found in one of the relief camps. As an ASHA Jennifer was covering villages with people belonging to her community.

 According to Jennifer, her husband and other men from the community were attending a peace meeting when the ethnic conflict started. She and her two daughters and sister-in-law were alone at home when they got to know about the conflict. Her youngest daughter was a week old new-born. Her daughters, sister-in-law and she ran for their lives and reached the first of many relief camps, never able to return home in native village for fear of further violence. Jennifer settled in a village that hosted relief camps during 1998 conflict.

While narrating her experience of fleeing for life, Jennifer mentioned that “*I took her [youngest daughter] for some time and I was unable to run properly and my elder daughter was also running. Everyone in our family ran and I was left behind. My sister-in-law helped me and took her [youngest daughter]. We stayed the night at XX [place and camp name withheld]. Her father [Jennifer’s husband] didn’t come and we couldn’t find him. We lost all our property and land. I stayed near the camp area and never returned home.*”

At the time of the interview, Jennifer expressed her fear of losing her job because households in her area were opting for family planning, hence there were fewer pregnant women compared to other villages. Given the thrust on institutional deliveries under a conditional cash transfer scheme known as Janani Suraksha Yojana, this incentive per pregnant woman was the main source of incentive for ASHAs. Jennifer who joined as ASHA in 2008, was afraid of losing her incentives and even worse, her job altogether:

*Now, I am working as an ASHA. What is ASHA’s salary? And there are no pregnant women in my area ma’am. Income is there in an area where pregnant women are there. The Government is asking to leave but where will I find another job?*

We observed that the health system was concerned about ASHAs performance in terms of rendering health services, meeting the planned targets, and being available whenever called for duty. We did not see, on the part of the health system, an investment in understanding or addressing the challenges and vulnerabilities faced by ASHAs as health workers and community members, even as these would directly affect their performance and the envisioned targets of the health system.

ASHAs like Jennifer had experienced conflict much the same way the community did. Their family members were killed, injured, tortured, or went missing, while others experienced psychosocial distress. ASHAs also suffered the effects of conflict during and after due to resource depletion and dislocation, just as other members of their communities.

## Discussion

This paper has focused on the experiences of four community health workers (CHW) working in conflict-affected parts of India and has demonstrated several challenges to the lives and livelihoods of the CHW. Notwithstanding this, all four ASHAs demonstrated resilience during the conflict and were continuing their work by overlooking the health system’s inability to recognize or respond to their vulnerabilities.

The situation of the geographic region we covered is unique. The concentration of conflict in certain districts – as well as the lack of recognition of conflict by the states in the region– has meant that ASHAs’ challenges and vulnerabilities in these situations and locations are yet to receive attention from state and federal policy-makers. In contrast, conflict in the state of Chhattisgarh has been recognised, Left Wing Extremist (LWE) areas have been designated such that services and special schemes are being delivered in these areas through academics and partnerships with a public purpose [20]. The role of civil society in areas with large tribal populations and conflicts such as Gadchiroli in Maharashtra has been significant [21]. We suggest Assam, like Chhattisgarh, to first recognize challenges and vulnerabilities that ASHAs working in these areas continue to experience. We suggest health systems to consider incentivising the ASHAs when they provide services during conflict.

Concurrently, there is a need to design a support mechanism for CHWs working in fragile settings. While designing this mechanism, the health system must be mindful that workers deserve adequate training, support, recognition, and compensation for the tasks they are carrying out in areas and situations where other cadres and workers are simply unavailable. We also suggest psychological support and job security for CHWs working in fragile and conflict settings, which has already been raised as an important need in outbreak contexts [22].

This would be an important step as our study and global literature suggests that in conflict settings, CHWs roles becomes more vital as they provide key links to the community and significantly outnumber formal health workers, often acting as the only serving, resident health care worker in insecure areas [23]. It is evident from the literature on human resources for health that CHWs are recognized as a critical cadre in the last mile or doorstep healthcare delivery in both conflict and non-conflict settings [1, 6, 22, 24]. For instance, in conflict-affected countries like Afghanistan, Sierra Leone, Liberia, and the Democratic Republic of Congo, CHWs played an important role in providing a basic package of health services to the community and managing outbreaks such as Ebola [6, 23, 25]. In cases of protracted conflict, as in Palestine and Lebanon, small scale CHW programmes have shown feasibility and some results [26, 27], although there is a need for more research in this area, in India and globally.

Similarly, there is a need to generate research evidence, to understand CHWs’ challenges and vulnerabilities globally in conflict settings. At present, the predominant concern of the global public health community working in fragile and conflict-affected settings is with delivering on targets – mostly related to contact coverage. This approach narrowly focuses on determinants of CHWs’ performance such as training, supervision and incentives, and supplies. Only a few studies have reported physical and emotional trauma, social, gender, and cultural norms faced by CHWs working in fragile and conflict settings [6, 28]. Gilmore et al. have reported that CHWs face safety and insecurity issues, disconnect from social systems, and under-resourced working conditions during the conflict, and despite these challenges had shown resilience [5].

Our study corroborates these findings: we found ASHAs showing tremendous courage and resilience in the face of hardship to provide institutional births and basic health services. Conflict not only weakened their efforts to transport the women to a health facility for childbirth, but it also broke social relations, particularly when the conflict was based on ethnicity. This increased distrust and indifference towards ASHAs in the community makes them vulnerable to violence and their security at threat. What is noteworthy to find that amidst fear and recognition of indifferent attitudes towards them, ASHAs continued to render services to the community and rebuilt the relationship with them over time. This, however, cannot be generalized as we believe there are narratives and experiences of ASHAs where they have had to bear the brunt of the breakdown of social relations and may have left the job or were reluctant to reach out to the community belonging to different ethnic communities.

Another key impact of conflict is displacement. Based on our findings and those of previous studies in fragile and conflict settings, it is evident that communities are vulnerable to displacement [29–32] and CHWs, being a part of the community, are no exception. We know that the displacement of populations is among the most severe consequences of contemporary conflicts. We have seen that ASHAs in our study settings had experienced both temporary and permanent displacement, because of the repeated conflicts. They are uprooted from their home and forced to settle in a new area. This is likely associated with psychological trauma and burnout. Gilmore et al. suggest that conditions for a good CHW programme include appropriate incentives, strong work factors such as training, supervision, security, and consideration for the individual and community context [5]. In the recent context of the COVID-19 pandemic as well, the safety and security of frontline health workers, as well as the stigma faced by them, have been raised as critical issues for the health system to address [22]. We suggest health systems to actively engage with existing structures in the villages such as village development committee, village health, and sanitation committee or peace committees to ensure safety and security of CHWs as well as establishing an environment for them to perform their tasks. In addition, health system must provide pick and drop transport facility to CHWs engaged in relief camps or any health service during conflict, as a measure to ensure their safety.

Concurrently, we suggest incorporating psychological first aid in the ASHA training to equip them with basic skills related to mental health and well-being. ASHAs we spoke to have no training or system support to deal with trauma, injury, mental health challenges, which are more prevalent and affecting a broader segment of the population she serves. The lack of attention to these dimensions poses a large human toll in terms of morbidity and mortality in conflict. We believe that the skill on psychological first aid will help ASHAs in coping with their stress, and anxiety as well as the community they are serving. Psychological first aid training was found to be an important skill during the Ebola crisis in Sierra Leone and had a positive impact [33]; closer to home, a study with lay health workers providing mental health counselling was found to have an impact, although the role of such training in the mental well-being of workers themselves was not explored [7]. Concurrently, we also believe that having access to psychosocial support services such as counselling during and after the conflict will help ASHAs to take responsibility for their lives and the community at large.

A review of the ASHA programme has also raised the specific gendered challenges faced by these women workers in having to carry out unpaid labour at work and home [34]. Another factor mentioned in prior work is unpaid labour or compensation for the services and expenses (such as travel) incurred by them [35]. Our findings suggest that CHWs are no exception and that these vulnerabilities are enhanced in conflict situations. The health system must be mindful of not replicating the inequitable gender norms that may lead to the exploitation of CHWs.

Critically, the health system’s emphasis and incentivisation on institutional births as well as family planning– although essential – also creates perverse situations where successful family planning results in fewer pregnancies and thus lower compensation and job insecurity for this cadre of workers.

Globally, conflict and violence are currently on the rise: as community members themselves, the role of CHWs as the first response in emergencies is vital. This is currently evident in the COVID 19 outbreak where CHWs are the first line of defense [22, 36]. For example, in India ASHAs are playing a pivotal role in reducing the impact of COVID 19 – they are undertaking contact tracing and monitoring of home quarantined persons. The World Health Organization suggests equipping, training, and preparing CHWs to provide critical care and help to ensure equity in health at the community level. The work of CHWs will contribute to a country’s effort to ensure health care for all during the crisis.

While this is important, we cannot negate the fact that it is time for the global public health community to recognize that CHWs are not only health workers but also as a member of the community with challenges and vulnerabilities of their own. To ensure the quality of health services in fragile and conflict settings it is important to be reflective of CHWs needs and design a robust support system including post-conflict recovery so that it provides a sense of security as well as reduces CHWs vulnerabilities.

### Limitations

We presented the experiences of four ASHAs and their situations may not be generalisable to ASHAs in other situations. Our aim, however, was to emphasise workers in these precise contexts as we feel their situation is less understood. Emphasis was instead placed on understanding the conflict related experiences of these workers in context and with depth, through corroboration with observations and literature review. What is more critically absent are the perspectives of ASHAs who may have left their work due to conflict. We were also not able to closely probe into the dynamics of ASHAs with other workers on the or at the facility and higher levels frontline (although in many cases, other workers were simply not there). Further research should also examine the roles and experiences of ASHAs in different types of conflict, ideally across Indian states with varying responses to the conflict to determine ways forward.

## Conclusions

 This is among the first studies in India examining the experiences of community health workers, specifically ASHAs, delivering care, and living in conflict situations. We noted the salience of simple social determinants of health, like transport, labour security, as well as the challenges of displacement and loss. The issue of trust – vitiated in conflict – was also noted. Conflict must be recognised as an important context within which community health workers operate, with greater policy focus and research devoted to understanding and addressing their professional and societal barriers, and greater system support to CHWs working in such conditions to enable them to perform their duty even in the face of the challenging situation.

## Data Availability

Not applicable.

## Abbreviations

ASHA: Accredited Social Health Activist
CHW: Community Health Workers
PHC: Primary Health Center
SC: Sub Centre
